# A Historical View of Motion Sickness—A Plague at Sea and on Land, Also with Military Impact

**DOI:** 10.3389/fneur.2017.00114

**Published:** 2017-04-04

**Authors:** Doreen Huppert, Judy Benson, Thomas Brandt

**Affiliations:** ^1^Institute for Clinical Neurosciences, German Center for Vertigo and Balance Disorders, Ludwig-Maximilians University, Munich, Germany; ^2^Institute for Clinical Neurosciences, Ludwig-Maximilians University, Munich, Germany

**Keywords:** seasickness, cart-sickness, litter-sickness, camel-sickness, Chinese antiquity, Greek antiquity, Roman antiquity, Red Cliff Battle

## Abstract

Seasickness and its triggers, symptoms, and preventive measures were well known in antiquity. This chapter is based on an analysis of descriptions of motion sickness, in particular seasickness, in ancient Greek, Roman, and Chinese literature. A systematic search was made from the Greek period beginning with Homer in 800 BC to the late Roman period and ending with Aetios Amidenos in 600 AD, as well as in the Chinese medical classics dating from around 300 AD. Major aspects are the following: body movements caused by waves were identified in all cultures as the critical stimuli. The ancient Greeks and Romans knew that other illnesses and the mental state could precipitate seasickness and that experienced sailors were highly resistant to it (habituation). The Chinese observed that children were particularly susceptible to motion sickness; they first described the type of motion sickness induced by traveling in carts (cart-sickness) or being transported on a litter or in a sedan chair (litter-sickness). The western classics recommended therapeutic measures like fasting or specific diets, pleasant fragrancies, medicinal plants like white hellebore (containing various alkaloids), or a mixture of wine and wormwood. The East knew more unusual measures, such as drinking the urine of young boys, swallowing white sand-syrup, collecting water drops from a bamboo stick, or hiding earth from the kitchen hearth under the hair. The Greek view of the pathophysiology of seasickness was based on the humoral theory of Empedokles and Aristoteles and differed from the Chinese medicine of correspondences, which attributed malfunctions to certain body substances and the life force Qi. Many sources emphasized the impact of seasickness on military actions and famous naval battles such as the Battle of the Red Cliff, which marked the end of the Han dynasty in China, or the defeat of the Spanish Armada by the English in 1588. A peculiar form of motion sickness is associated with Napoleon’s camel corps during the Egyptian campaign of 1798/1799, a sickness induced by riding on a camel. Thus, motion sickness in antiquity was known as a physiological response to unadapted body motions during passive transportation as well as a plague at sea.

## Introduction^1^

Travel by sea was an important development in the history of mankind, for with it trade began on an even wider scale than was possible on land. Professional sailors were in demand; however, as the classical authors East and West inform us, not everyone was suited. Ruler and ruled alike could suffer from the so-called “plague at sea,” i.e., seasickness. The clinically most relevant facts about seasickness were already described in the Greek and Roman classics: triggers and susceptibility, symptoms, preventive measures, and therapy (1). The Chinese medical classics distinguished several forms of travel sickness, and each form had its own written character (2).

Medical knowledge of the times was initially limited and typically dominated by fears and superstitions. In the West, the Greek *Empedokles* was the first to propose a theory to explain human nature and illnesses: the four humors or body fluids (yellow bile, phlegm, black bile, and blood) had to be in equilibrium for a harmonious personality and health. Any imbalance led to deviation from normal and to illness. The theory continued to be influential into medieval times. In particular, it was the primary source for Greek, Roman, and medieval explanations of seasickness. In the East, belief in magic and charms lasted longer, perhaps because of the dominating quasi scientific theory of the medicine of correspondences. This theory connected climatic phenomena, cardinal directions, and calendric constellations with the actions of specific forces in the world and with the supply of the life force (Qi) and blood (xue) in the human body.

## Historical Text Sources

Beginning with the works of *Homer* (ca. 800 BC) and ending with the work of *Aetios Amidenos*, we searched Greek and Roman texts up to 600 AD (Table 1). This search focused on terms like “ναυτία, ναυσία, ναῦς, nausea, navis” and their linguistic derivatives such as “ναυτιάω, ναυσιάω, ναυσιάσις, ναυτιῶντι, nauseare, nautea” (see Etymology and Terminology) in the dictionaries Totius Latinitatis lexicon (3), Georges (4), and Thesaurus Linguae Latinae (5) as well as in the commentaries on the citations and cross-references. The most important original sources included texts by *Hippokrates, Aristoteles, Plautus, Cicero, Celsus, Seneca Pater and Seneca Minor, Plinius, Plutarchos, Ovid, Juvenal, Lucian, Philostratos, Gellius*, and *Galenos* (see Table 1 in which all classical sources cited are listed in alphabetical order by author). Furthermore, secondary literature on the history of seasickness (6–10) and a comprehensive doctoral dissertation on seasickness in antiquity (11) were searched.

**Table 1:** **Classical Roman and Greek sources describing seasickness, alphabetized by author [mod. after Huppert et al. (1)]**.

Author	Biographical data	Opus	Text source/	Publisher
**Aetios Amidenos**	6th c. AD	**Logon iatrikon biblioi**	Corpus Medicorum Graecorum, Vol VIII, 1. Oliveri A. (ed.)	Leipzig, Berlin, 1935
Aëtios of Amida		Medical books		

**Aretaeus Cappadocus**	81–138 AD	**Opera quae supersunt**	Therapeutics of chronic affections. Book II. Adams F. (ed., trans.)	Printed for the Sydenham Society, London, 1956
		The extant works		

**Ariston Chiou** Ariston of Chios	2nd c. BC	**Stoicorum veterum fragmenta**	Bd. I, Zeno et Zenonis discipuli. V. Arnim H. F. (ed)	Leipzig, 1905
		Ancient stoicism: the editions of fragments and *testimonia*		

**Aristoteles** Aristotle	384–322 BC	**Problemata physika**	Flashar H. (ed., trans.)	Wissenschaftliche Buchgesellschaft, Darmstadt, 1962
		Problems		

**Athenaios** Athenaios of Naukratis	Late 2nd c. AD	**Deipnosophistai**	Book XV, Garlands (Loeb Classical Library 519) Olson S. D. (ed., trans.)	Harvard University Press, Cambridge, London, 2012
		Banquet of the learned		

**Caesar (Caius Julius)**	100–44 BC	**Bellum civile**	Book III (Loeb Classical Library Vol 39). Peskett A. G. (ed., trans.)	Heinemann, London, The Macmillan Co., New York, 1914
		The civil war		

**Celsus (Aulus Cornelius)**	25 BC–50 AD	**De Medicina**	Vol. I (Loeb Classical Library 292). Spencer W. G. (ed., trans.)	Heinemann, London, Harvard University Press, Cambridge, 1935
		On medicine		

**Cicero**	106–43 BC	**Epistulae ad familiares**	Vol. III, Epistulae ad familiares, XVI.XI. (Loeb Classical Library Vol 230N). Williams W. G. (ed., trans.)	Heinemann, London, Harvard University Press, Cambridge, 1940
		Letters to his friends		

**Galenos**	129–199 AD	**Hippokratou aphorismoi kai Galenou eis autous hypomnemata**	Vol XVII,1,2; XVIII,1. Claudii Galeni opera omnia. Kühn C. G. (ed)	Leipzig, 1829
		Aphorisms of Hippocrates and Galen with commentaries		

**Gellius Aulus**	125–180 AD	**Noctes Atticae**	Auli Gelli Noctes Atticae cum indicibus locupletissimis	Tauchnitz, Lipsiae, 1835
		Attic nights		

**Hesychios Alexandrinos** Hesychios of Alexandria	5th/6th c. AD	**Synagogae pason Lexeon kata Stoicheion**	Hesychii Alexandrini Lexicon, Schmidt M. (ed.)	Jena, 1867
		Alphabetical collection of all words		

**Hippokrates**	460–370 BC	**Aphorismoi**	IV.VIII.-XV. (Loeb Classical Library Vol 150)	Heinemann, London, Harvard University Press, Cambridge, 1959
		Aphorisms	Jones W. H. S. (ed., trans.)	

**Homer**	8th c. BC	**Odysseias**	Vol. I,V.304–330; Vol I, V. 442–466 (Loeb Classical Library Vol 104)	Harvard University Press, Cambridge, Heinemann, London, 1945
		The Odyssey	Murray AT (ed., trans.)	
		
		**Odysseias**	Schadewaldt W. (ed., trans.)	Artemis Verlag, Zürich, 1966
		The Odyssee		

**Horatius** Horace	65–8 BC	**Epistulae**	I.I.62–85 (Loeb Classical Library Vol 194). Rushton Fairclough H. (ed., trans.)	Heinemann, London, Harvard University Press, New York, 1942
		Epistles		

**Juvenal**	60–130 AD	**Saturae**	Satire VI (Loeb Classical Library Vol 91N). Ramsay GG (ed., trans.)	Heinemann, London, Putnam’s Sons, New York, 1928
		Satires		

**Livius Titus**	59 BC–17 AD	**Ab urbe condita**	Lat.: Book XXI, 25	Oxford University Press, 1929 (lat.), 2006 (engl.)
		Hannibal’s War	Engl.: Book 21, chapters 25–27	

**Lucian**	120–180 AD	**Charon**	Vol. II (Loeb Classical Library Vol 54). Harmon AM (ed., trans.)	Heinemann, London, Putnam’s Sons, New York, 1919
		
		**Toxaris**	Vol. V (Loeb Classical Library Vol 302). Harmon AM (ed., trans.)	Heinemann, London, Harvard University Press, New York, 1962
		
		**Hermotimus** or the rival philosophies	Vol. VI (Loeb Classical Library Vol 430). Kilburn K (ed., trans.)	Heinemann, London, Harvard University Press, Cambridge, 1949

**Macer, Aemilius (Pseud.)**		**De viribus herbarum**	Choulant L (ed., trans.)	Leopoldi Vosii, Leipzig, 1932
		About Herbal Plants		

**Martial**	40–104 AD	**Martialis Epigrammata**	Book IV.XXXVII (Loeb Classical Library Vol 94). Ker WCA (ed., trans.)	Heinemann, London, Putnam’s Sons, New York, 1919
		Epigrams		

**Oreibasios**	325–403 AD	**Oreibasiou iatrikai synagogai**	Corpus Medicorum Graecorum, Vol VI.1.1. Raeder I (ed.)	Leipzig, Berlin, 1928
		Collection of medical relics		
		
		**Oreibasiou synopsis pros Eustathion ton huion**	Corpus Medicorum Graecorum, Vol VI,3. Raeder I (ed.)	Leipzig, Berlin, 1926
		Synopsis to Eustathium		

**Ovid**	43–18 AD	**Amores**	The remedies of love (Loeb Classical Library Vol 232). Mozley J. H. (ed., trans.)	Heinemann, London, Harvard University Press, Cambridge, 1957
		The Art of Love, and Other Poems		

**Petronius**	14–66 AD	**Satyricon**	(Loeb Classical Library Vol 15). Peskett M. (ed., trans.)	Heinemann, London, The Macmillan Co., New York, 1913

**Philostratos**	170–247 AD	**Ta es ton Tyanea Apollonion**	Vol. I, book IV (Loeb Classical Library Vol 16N). Conybeare F. C. (ed., trans.)	Heinemann, London, The Macmillan Co., New York, 1912
		The life of Apollonius of Tyana		

**Plato**	428/7–348/7 BC	**Nomoi**	Vol. I, Laws, book I (Loeb Classical Library Vol 187). Bury R. G. (ed., trans.)	Harvard University Press, Cambridge, Heinemann, London, 1940
		Laws		

**Plautus**	255–184 BC	**Amphytrion**	Vol I (Loeb Classical Library Vol 60). Nixon N. (ed., trans.)	Heinemann, London, Putnam’s Sons, New York, 1916

**Plinius** (the Elder)	23–79 AD	**Historiae naturales**	Vol VI, libri XX, XXI and Vol VII, liber XXVII (Loeb Classical Library Vols 392, 393). Jones W. H. S. (ed., trans.)	Harvard University Press, Cambridge, Heinemann, London, 1951, 1956
		Natural History		

**Plutarchos**	40–120 AD	**Moralia. Aitiai physikai**	Vol XI (Loeb Classical Library Vol 322). Pearson L, Sandbach F. H. (trans.), Capps E. (ed)	Heinemann, London, Harvard University Press, Cambridge, 1965
Plutarch		Natural phenomena		
		
		**Moralia. Hygieina parangelmata**	Vol. II, 86B-171F., 126 (Loeb Classical Library Vol 222). Babbitt FC (ed., trad.)	Harvard University Press, Cambridge, Heinemann, London, 1962
		Advice about keeping well		
		
		**Politika parangelmata**	Vol X, 798 (Loeb Classical Library Vol 321). Fowler H. N. (trad.), Warmington E. (ed.)	Heinemann, London, Harvard University Press, Cambridge, 1969
		Precepts of statecraft		

**Pseudo-Apuleius Platonicus**	4th c. AD	**Herbarius**	Corpus Medicorum Latinorum, Vol IV. Howald E, Sigerist H. E. (eds.)	Leipzig, Berlin, 1927
		Herbarium		

**Semonides**	7th/6th c. BC	**Iamboi**	Anthologia lyrica Graeca 3: Iamborum scriptores. Diehl E. (ed.)	Teubner, Leipzig, 1952

**Seneca Pater**	54 BC–39 AD	**Suasoriae**	(Loeb Classical Library Vol 464). Edward W. A. (ed., trans.)	Harvard University Press, Cambridge, New York, 1928
Seneca the Elder		The Suasoriae of Seneca the Elder		

**Seneca Minor**	4 BC–65 AD	**Ad Lucilium Epistulae Morales**	Vol. I, Epistle LIII (Loeb Classical Library Vol 77). Gummere R. M. (ed., trans.)	Harvard University Press, Cambridge, Heinemann, London, 1979
Seneca the Younger		The Epistles of Seneca		

**Soranos**	2nd c. AD	**Peri gynaikeion**	Lüneburg H. (ed., trans.), Huber J. C. (ed.)	Lehmann, München, 1842
		On Midwifery and the Diseases of Women		

**Theophrastus**	371–287 BC	**Peri phyton historias**	Wimmer F. (ed.)	Paris, 1866
Theophrast		Enquiry into plants		

To analyze the Chinese historical texts, we first searched primarily for specific terms for seasickness in the Internet and in modern dictionaries (12). The dictionaries indicate which Chinese characters are used and cite classical works in which they appear. There have always been attempts throughout the Chinese tradition to record and summarize large encyclopedic works, the last and most important of which is the Siku quanshu (Encyclopedia in four parts) from 1782. In a second step, the different words used for seasickness were entered into the most important databases of full texts, allowing selection of relevant text passages. The immense written tradition of Chinese culture is, nowadays, readily accessible in databases like Scripta Sinica, The Complete Classics Collection of Ancient China, The Treasure of National Culture, the Siku quanshu, The Chinese Text Project, and Dissertations of China (Table 2). We concentrated on the characters for the different types of dizziness: ship-influence, cart-influence, and wave-influence. Search words included the various concepts of dizziness: (1) 注船 zhuchuan, ship-influence, (2) 注車 zhuche, cart-influence, (3) 疰船 zhuchuan, ship-influence (different character), (4) 注浪 zhuliang, waves-influence, (5) 暈車 yunche, cart-dizziness, (6) 暈船 yunchuan, ship-dizziness, (7) 苦船 kuchuan, ship-illness, (8) 苦车 kuche, cart-illness, and (9) 船疰 chuanzhu, ship-influence (different character). The third part of the search involved the analysis of relevant text passages, in particular the sea battle of Chibi, during which seasickness evidently played a decisive role. See Table 3 for a chronological list of citations from the original Chinese works.

**Table 2:** **Important databases of the written tradition of ancient Chinese culture [mod. after Brandt et al. (2)]**.

**Scripta Sinica**: the largest Chinese full-text database with an enormous breadth of historical materials. More than 460 titles and 402,000,000 characters of materials pertaining to the traditional Chinese classics are categorized. It contains almost all of the important Chinese classics, especially those related to Chinese history

**The Complete Classics Collection of Ancient China: The Gujin tushu jicheng** 古今圖書集成, compiled by Chen Menglei and co-workers. It was published with movable copper type in 1726–1728 AD and is the largest reference book historically compiled in China (*leishu)* to have been printed (6,109 subsections; 852,408 pages). Each subsection contains excerpts from sources dating from the beginnings of writing to the 17th century AD. All were selected from an eighteenth-century standpoint

**The Treasure of National Culture: The Guoxue baodian** 国学宝典 is the largest volume in Chinese classical literature and covers most varieties of subjects in Chinese classical literature. It contains a database of more than 50,000 poems from all epochs, a number of notebooks 笔记, theater pieces, and novels (白话 and 文言). By 2012 AD, the database contained more than 4,000 titles

The encyclopedia **Siku quanshu**: it provides an electronic database published by the Chinese University of Hong Kong and Digital Heritage Publishing Ltd., consisting of the complete contents, in full text and images, of the 3,460+ titles of the Wenyuange edition of the **Siku quanshu** (1,782) plus a few other useful tools. The staggering 800 million characters are arranged by almost 2 million titles

**The Chinese Text Project**: an ancient text database containing the full text of various Chinese texts of philosophical, historical, or linguistic interest from the pre-Qin era through to the Han dynasty and beyond. http://ctext.org/

**Dissertations of China**: it collects dissertations, master theses, and post-doc papers from PRC universities, graduate schools, and research institutes. Starting with the year 1980 and as of the year 2012, about 2.3 million qualification works had been provided in searchable full text

**Table 3:** **Chronological overview of relevant text passages in the original Chinese works on cart-sickness and seasickness [mod. after Brandt et al. (2)]**.

Time	Concept	Works	Author
ca. 300 AD	Cart-influence and ship-influence (注車注船, zhuche zhuchuan, literally, influence-cart, influence-ship)	**Zhou hou bei ji fang**	Ge Hong (281–341)
		肘后备急方 (emergency remedies/formulae to carry behind the elbows)	葛洪;

610 AD	Cart-influence and ship-influence	**Zhu bing yuan hou lun**	Chao Yuanfang
		诸病源侯论 (discussion about signs and causes of all illnesses)	巢元方 (ca.550–630)

725~777 AD	苦车 Cart-illness (literally, bitterness-cart)	Poem: 《癸卯岁赴南丰道中闻京师失守寄权士繇韩幼深》	Du Guji
			独孤及 (725~777)

983 AD	苦车Cart-illness (literally, bitterness-cart)	**Taiping yulan**	
		太平御覽, (imperial notes from the Taiping era); *juan* 918 卷九百一十八.羽族部五 (found in **Gujin tushu jicheng**)	

12th century AD	Ship-illness, cart-illness (苦船 kuchuan 苦车 kuche; literally, illness-ship and illness-cart)	**Xi xi cong yu** 西溪叢語 (collection of talks about the Western stream)	Yao Kuan
			姚寛 (1105–1162)

14th century AD	注船 Ship-influence	**Danxi Xinfa** 丹溪心法 (core of the teachings of [Zhu] Danxi)	Zhu Danxi
			朱丹溪 (1281–1358)

1578 AD	注車注船 Cart-influence and ship-influence	**Ben cao gang mu** 本草綱目 (Compiled medical material); Section Herbs 草部, 10th *juan* 凡十卷/第十三卷/草之二山草類下三十九種/徐長卿 (p 822) refers to the entry for the herbal substance Xú Zháng Qīng 徐長卿 RADIX CYNANCHI PANICULATI, Paniculate Swallowwort Root	Li Shizhen
			李時珍 (1518–1593)

1596 AD	注船 Ship-influence	**Chi shui yuan zhu** 赤水元珠 (Pearls of wisdom lifted out of the purple sea); Cited in **Siku quanshu**, Section on Medicine, 9th *juan*, 子部/醫家類/赤水元珠/卷九	Sun Yikui
			孙一奎 (1538–1600)

17th/18th century AD	Ship-dizziness and cart-dizziness (晕船晕车 yunchuan yunche, literally, dizziness-ship and dizziness-cart)	**Yan fang xin pian** 验方新编 (New collection of proven remedies); *juan* 16, various treatments [卷十六\杂治], ship-dizziness-cart-dizziness晕船晕车	Bao Xiang’ao
			鲍相璈

18th century AD	注船注轿 Ship-influence and litter-influence	**Tong su bian** 通俗编 (Notebooks on miscellaneous things); *juan* 26.(Zhai Hao 翟灏, **Tong su pian**《通俗编》-卷二十六) (in **Guoxue Baodian**)	Zhai Hao
			翟灏 (?–1788)

## Historical Theories of the Mechanisms Underlying Seasickness

### Humoral Theory—From Greek and Roman to Medieval Times

The classical authors tried to explain disorders in general within the framework of the humoral theory of *Empedokles*, which was valid at that time: the four body fluids (“χολή” = yellow bile, “φλέγηα” = phlegm, “μέλαινα χολή” = black bile, “αἷμα” = blood) were said to be at such times out of balance (13). *Aristotle* was the first to explain seasickness on the basis of the humoral theory. One of his students and eventual successor, the philosopher *Theophrastus* also used the humoral theory to analyze seasickness. Later, the Roman stoic philosopher *Seneca the Younger*, a medical layman, delved deeper. He localized seasickness in the stomach. The Latin “stomachus” or the Greek “στόμαχος” at that time referred not only to the anatomic organ of the stomach but also to the esophagus. For *Seneca*, an important feature of seasickness was the bile vomited by the seasick. He graphically described a bout of seasickness as “a convulsion of the bile.”

Medieval European medicine observed correspondences between the heavenly bodies, the fluids in the human body, the temperaments, and illnesses by means of which the phenomena of the macrocosm (world) and the microcosm (man) were ordered and divined.

### Chinese Medicine of Correspondences and Ancient Magic

A passage from the book **Chi shui yuan zhu** (Pearls of wisdom lifted out of the purple sea) by *Sun Yikui* from 1596 shows a similar interest in bile (14):

“Later due to seasickness she vomited up a number of bowls of saliva, her condition did not improve, it originated in her observation of [her own] vomited blood and in her condition of liver stagnation.” However, the dominating feature of Chinese medicine is Qi, the body’s life force. If it flows incorrectly or is agitated by emotions, problems arise. For example, liver stagnation is said to occur due to an incorrect flow of Qi—the life force in the body which is generally triggered by such internal factors as emotional agitation.

In contrast to the Greeks and Romans who focused on the stomach as the site of the causes of dizziness, the Chinese emphasized the liver and the eyes. They drew attention to the interconnection between the liver and eyes already in the 4th century BC, but interpreted dizziness to be an optical phenomenon and thus a malfunction of the eyes. The most important Chinese character for dizziness was 眩 xuan, which consists of the phonetic character xuan on the right and a semantic indicator “eye” on the left. This character stands for the semantic field of darkness, black, unsureness, but also suggests the pronunciation of the character. Thus an image is evoked of how it becomes dark before the eyes during dizziness. The dictionaries indicate that the character refers to different types of dizziness which were never really differentiated from each other. The early dictionaries [Shuowen Jiezi, ca. 1st century (15)] use the character xuan to refer to visual malfunction or movement and chaos.

Based on the systematic correspondences of specific forces in the macro- and microcosm, the quasi-scientific Chinese medical theory is called a medicine of correspondences. Dizziness is a good example of how the Chinese understood this term: certain elements in the macrocosm like wood and wind were connected with the organs of the liver and the eyes of the microcosm. The following often repeated citation comes from **Huangdi Neijing Suwen** (15): “[diseases with] wind [that causes] tumbling and dizziness belong without exception to the liver.” Thus, in the case of optical phenomenon, the liver is immediately considered the cause.

Another organ (in our modern meaning) that was associated with the phenomenon of dizziness is the brain. Although the brain plays no role in Chinese medicine, belonging neither to the yin organs (heart, spleen, lungs, kidneys, liver) nor the yang organs (small intestine, stomach, large intestine, bladder, gallbladder), it was apparently considered an organ in which the substance “marrow” was stored. Marrow is another word for the spinal cord. Thus, in one of the oldest and most fundamental texts of Chinese medical theory, the Yellow Emperor’s Inner Canon—Axis of the forces (Huangdi Neijing Lingshu (15)), there is a description of height intolerance: a pathogen is said to enter through the neck, make the brain roll around, and as a result the interconnection to the eyes becomes taught, causing dizziness. This suggests there was anatomical knowledge of a connection between the brain and the eyes. It is interesting that this very clear and mechanistic description of the cause of dizziness was taken over also in later works. It apparently was based on a theory of anatomy, which was not thematized thereafter. In general, the ancient Chinese with very rare exceptions never investigated the anatomy of the human body.

Different scholars and physicians have made various subdivisions, for example, essences (jing) and blood (xue) were conceived as implementations or various aggregate conditions of the same life force Qi. Assertions about the significance of these substances in connection with dizziness appear in the earliest sources:

If Qi is insufficient above, the brain is not sufficiently filled by it, the ears suffer a ringing noise, the head is bent low by it, the eyes [experience] dizziness.

Here the brain is to be understood as the “sea of marrow,” i.e., the storage site of the marrow—a body substance. Thus, dizziness occurs in connection with tinnitus and an imbalance of the head due to deficient Qi in the head (16). The following citation from the chapter “Discussion of the sea” refers to the same subject matter: “Is the sea of marrow not sufficiently filled, the brain begins rotating around, the ears ringing, the calves/shins suffer from sour pains and the veil-dizziness (xuanmao) appears, the eyes can see nothing and passivity and hypersomnia occur.” Besides a deficit of Qi in the head, numerous other sick conditions of dizziness, which can be ascribed to a lack or surplus of substances or “energies” in the body, are described: “dizziness due to increasing fire,” due to phlegm, to a deficit in yin, a deficit in yang, a surplus of yang, and a blood deficit.

## Etymology and Terminology

### Greek and Roman Times

In Greek antiquity, the term used to express the experience of seasickness (nausea) was based on the word for ship: “ναῦς” [naus]. The corresponding verb meaning “I am seasick” was “ναυτιάω” [nautiao] or “ναυσιάω” [nausiao]. The Latin words “nausea” and “nauseare” were derived from the Greek. The meaning of these words had been amplified already in antiquity: besides seasickness they also stood for a general sick feeling and vomiting as well as disgust and tedium (11). Examples of such amplification and diversification of the original meaning can be found in early texts of the Greek poet *Semonides*, who in the 7th/6th century BC already used “ναυτία” to describe such a sick feeling. In the 5th/6th century AD *Hesychios Alexandrinos* presented an alphabetical dictionary on these different meanings of “ναυτία” and “ναυσία” and their derivatives. The connotation of seasickness in the word for sea travel was subsumed by the term “ναυτίῶντι” [nautionti], which he paraphrased with the words “κλύδωνι περιφερομένῳ” [klydoni peripheromeno]. The latter means “he whom the waves of the sea make dizzy.” Like many things in Greek culture, the Romans took over these multiple meanings. For example, the poet *Ovid* used “nausea” when describing a revolting meal and later *Martial* also used it to suggest disgust. Such multiplicity recalls the results of our search for the first descriptions of fear of heights in Greek and Roman antiquity. The Latin word “caligo” had multiple meanings: vertigo as a symptom occurring in the context of heights, but also metaphorically, as evoking feelings of exultation (17).

**Figure 1:**
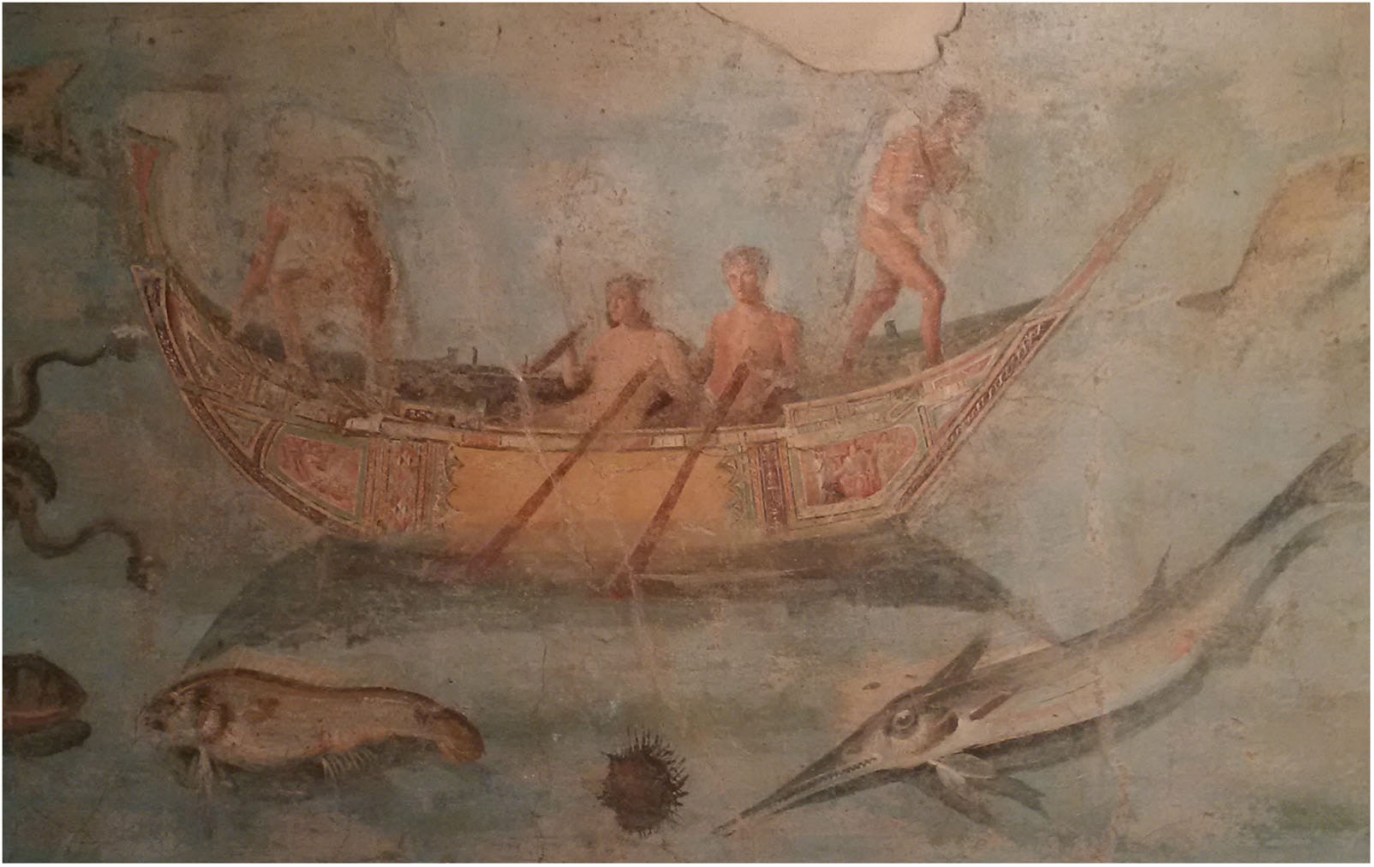
**Roman rowing boat**. Fragment of a wall painting of marine life from the Museo Nazionale Romano, Palazzo Massimo alle Terme, Rome (Dalla località Pietra Papa presso il Porto Fluviale di San Paolo lungo la via Portuense. 125–150 AD; private photograph Doreen Huppert).

Despite all of these multiple meanings for antiquity, motion sickness was mainly connected with the sea and ships. Beginning with the First Punic War in the second century BC (18), marine navigation became more important in classical antiquity. Many different sizes of boats were used (Figure 1), not only for trade but particularly for warfare.

### Ancient China

Whereas the Greeks had observed the effect of the stimulus marine travel on individuals (cause and effect thinking), the ancient Chinese focused on the type of transport: ship, cart, etc. Only in modern times did this focus change. In ancient Chinese literature, for example in **Zhou hou bei ji fang** (emergency prescriptions/remedies to carry behind the elbow, around ca. 300 AD), the terms cart-influence (注车, zhu che) and ship-influence (注船, zhu chuan) are found. This first reference to illnesses due to motion is discussed by the author, a famous physician and alchemist, in a chapter on remedies to protect someone from evil forces, i.e., the influence of demons and corpses.

The poet *Du Guji* 独孤及 (725~777 AD) wrote a poem in which he uses the term cart-sickness (literally cart-bitterness). This poem can only be understood in the historical context of the collapse of the kingdom a few centuries earlier into a northern and a southern realm. In the early 3rd and 4th centuries AD, many aristocrats fled from the northern barbarians, traveling south into the inaccessible mountainous regions in the province of Jiangzi.

长叹指故山，三奏归来词。不逢眼中人，调苦车逶迟。

With a profound sigh they point to the unexpected mountains, already having thrice sung the Gulai Song. One will not see again those close to us, we move on, travel sick and weak ….

The work **Taiping yulan**, imperial records from the Taiping Era, an encyclopedia from the year 983 AD, refers to an alleged story from the records of Jin (Jin shu). The story is about an official named Chi Shen whose mother had a cart-illness. After she dies, he decides not to return her to her village with the cart, out of filial respect, and works for three years, sowing and reaping grain and raising chickens to earn enough money to transport the coffin home in style with eight horses and finally bury his mother. With slight variations, this story is found on Chinese internet sites as a copy of Jin (Jin shu, 九家舊晉書輯本).

The non-medical work **Xi xi cong yu** (notebooks) from the 12th century has an entry mentioning that people in the south use the term ship illness (苦船kuchuan), while people in the north say cart illness (/苦车 kuche).

今人不善乘船，谓之苦船；北人谓之苦车。

If people are not good at traveling by ship, it is called ship illness. People in the north call it cart illness.

**Figure 2:**
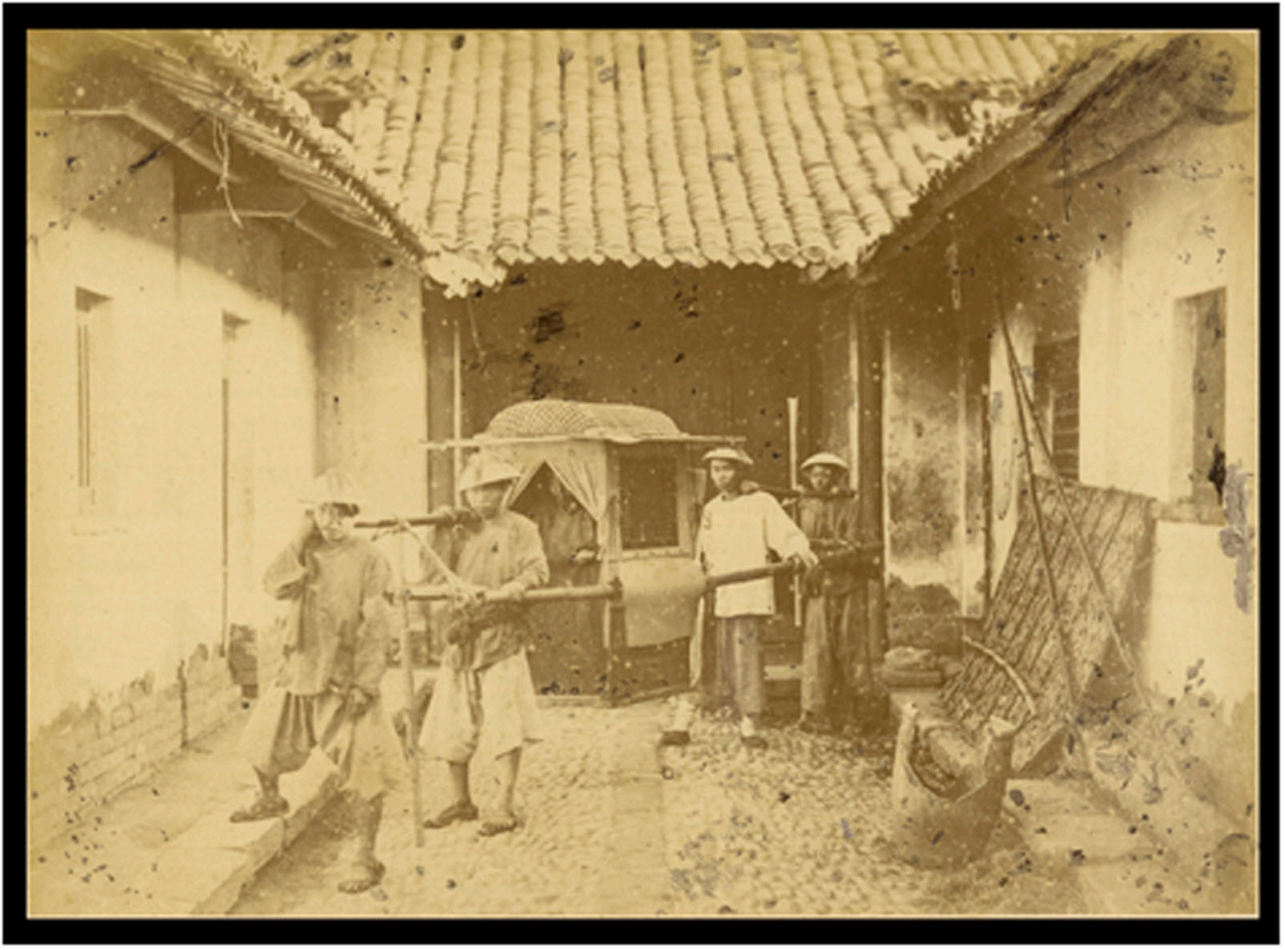
**An ancient form of human transportation**. A litter was a vehicle without wheels which porters carried by means of wooden rails (see photograph). The cabin of such a litter, also called a sedan chair, could be enclosed for protection from the elements. According to our current understanding of the pathomechanism of motion sickness, this means that while the vestibular system sensed the movements during transportation, the visual system received no information about motion. Such a sensory mismatch is known to facilitate the occurrence of motion sickness. The Chinese called this “litter sickness” or “litter influence” (photograph: sedan chair, China, 1874/75; https://commons.wikimedia.org/wiki/File:Sedan_Chair_Carried_by_Four_Men._China,_1874-75_WDL1930.png).

The terms dizziness ship yunchuan (for seasickness) and dizziness cart yunche (for travel illness with the cart) (晕船晕车 yun chuan yun che) are common usage in **Yan fang xin pian** from the 17th/18th centuries and are still standard today. This alteration of the terminology is also seen in the fact that old citations continued to be used with the additional clarification “Today one calls this dizziness ship (yunchuan).” In the notebooks of *Zhai Hao* entitled **Tong su bian** (notebooks on miscellaneous things) there is in juan 14 in the chapter “Collection of the nunnery Pin Luo” an explanation of the word ship-influence–litter-influence. This is the sole proof for the concept litter-influence, but it is clear that the swaying movements can lead to a travel illness similar to that of seasickness. A litter is a conveyance for transporting persons who sit on a bed or couch suspended between two long poles and are thus carried (Figure 2). Such vehicles were not only used in China (jiao or wo) but also in ancient Rome (lectica or sella), pharaonic Egypt, and various other countries throughout the centuries (it was called a sedan chair in Britain). As the nascent investigation of Chinese medical manuscripts shows, the less-educated social classes resorted more often to therapeutic concepts from the realm of demons and other magic and religious beliefs, as was seen in the concepts of cart-influence and ship-influence (see above). These manuscripts, however, are not available in printed form and could not be evaluated for our study. The entry in **Xi xi cong yu** was quoted in numerous later works and continued to be handed down. In many versions, the quote reads “persons from the south do not travel well by ship, they have ship-illness, persons from the north call the illness cart-illness.” This may actually be related to the fact that the rivers in southern China play a much more significant role in transport and travel than in the more arid north.

The modern term for motion sickness in China is 运动医 (yundong bing). It can be subdivided into car sickness, train sickness, or also travel illness 晕车 (yunche, literally dizziness-car) and seasickness 晕船 (yunchuan, literally dizziness-ship) (19). Various terms have been used in the Chinese written tradition to describe this phenomenon. The current term “yunche yunchuan,” i.e., travel illness and seasickness, first appeared in the 17th/18th centuries.

## Historical Knowledge of Triggers, Symptoms, Susceptibility, and Mental State

### Precipitating Stimuli

Several ancient Roman and Greek authors reported how wave-induced body motion was a predominant trigger and provoking factor of seasickness. According to *Horatius* (**Epistulae**), the ship size appeared to be of no importance for the development of seasickness. The medical layman *Caius J. Caesar* referred to a causal connection between the high undulations of the open sea and seasickness in his **De bellum civile**. This was later emphasized by the Greek physician *Galenos* in his **Hippokratou aphorismoi**. *Oreibasios* (**Oreibasiou iatrikai synagogai**) cited *Antyllos* from the first century AD, who claimed that particularly pitching and tossing are triggers at sea. They occur less often near the shore where also seasickness is less frequent. This was confirmed by *Plutarchos* in his **Aitiai physikai**: seasickness is reported less often on rivers than on the open sea. The latter also mentioned unpleasant odors as provocative stimuli for seasickness in susceptible persons:

**ἐν δὲ τῇ θαλάττῃ τήν τ’ ὀσμὴν ἀηθείᾳ δυσχεραίνουσι καὶ φοβοῦνται, μὴ πιστεύοντες τῷ παρόντι, περὶ τοῦ μέλλοντος•**

At sea, on the other hand, men find the smell disagreeable because of its strangeness, and not trusting the present weather to last, are anxious about what the future holds.

This was supported by *Caesar* and *Juvenal*, who also mentioned the “sickening smell” of bilgewater as a provocative factor.

While the Greeks primarily focused on the external world as the source of triggers, the Chinese, in accordance with their medical theory, basically emphasized internal imbalances or deficits of vital substances in the body. The external world, however, was also influential as the source of pathogens that could upset the body’s harmonious balance. The search for the causes of symptoms like dizziness, height intolerance, or motion sickness took place in ancient China within the prevailing view of the body. In the case of dizziness, the external pathogen was primarily the wind, which was generally associated with movement. It was assumed that the wind could penetrate a weakened body and induce symptoms and cause illness. This was then reflected in the body by disordered movements and sudden appearances. The wind was described as an especially aggressive force that gets everything moving (tumbling and falling) and can lead to chaotic conditions in the world and to illness in the human body. It is also instrumental in promoting the entry of other factors such as heat, moisture, and cold. Its connection with the liver, which was considered to play an important role in the storage of blood, could play havoc with the body. Blood is basically a material, and if there is a deficit of blood, the more mutable features of Qi cannot connect with it, thus leading to symptoms of an internal wind, which expresses itself in dizziness, tics, and twitches.

### Symptoms

Several classical authors describe the various symptoms of a manifest seasickness. In his biography (**Ta es ton Tyanea Apollonion**), *Philostratos* mentioned the prodromal symptoms of seasickness: these included the inability to maintain a conversation, the absence of concentration, and a certain indifference.

**…οὐ γὰρ ναυτιῶν γε, ἢ ὑπὸ τοῦ πλοῦ πονηρῶς ἔχων ἀποστρέφη τοὺς λόγους, ἡ γὰρ θάλαττα, ὁρᾷς, ὡς ὑποτέθεικεν ἑαυτὴν τῇ νηὶ καὶ πέμπει**.

For I am sure that it is not because you are seasick or in any way inconvenienced by the voyage, that you object to our conversation; for you see how smoothly our ship is wafted over her bosom by the submissive sea.

Bodily symptoms like nausea and vomiting are reported by, among others, *Petronius* and *Aretaeus*. The desiccative effects of vomiting were interpreted also in terms of a possible therapeutic effect for conditions like cephalalgia, renal diseases, or elephantiasis. *Juvenal* vividly described how vertigo is sometimes manifested:

**… tunc sentina gravis, tunc summus vertitur aer**.

… the bilge-water then sickens her, the heavens go round and round.

Due to nausea, seasick persons are anorectic, have no desire for cooked, boiled, or fried food according to *Plutarchos* in his **Hygieina parangelmata**. Instead, they crave salty food, even if they later have to disgorge it. In modern medical terms, this suggests an electrolyte imbalance. Other authors, such as *Homer* and *Gellius*, stress the occurrence of faintness, apathy, and abulia during seasickness. A passage in *Homer*’s **Odysseias** evokes the physical state of Ulysses, washed up on the beach:

**…ὁ δ‘ ἄρ‘ ἄπνευστος καὶ ἄναυδος κεῖτ‘ ὀλιγηπελέων, κάματος δέ μιν αἰανὸς ἵκανεν**.

…So he lay breathless and speechless, with scarce strength to move; for terrible weariness had come upon him.

Further, the association of anxiety and seasickness was recognized quite early. *Plato* was the first to emphasize this:

**…κἂν δειλὸς ὢν ἐν τοῖς δεινοῖς ὑπὸ μέθης τοὺ φόβου ναυτιᾷ;**

… even though he be a coward and sea-sick with a kind of tipsy terror when danger comes?

In addition, *Gellius* mentions paleness of the face as a symptom arising together with anxiety. *Lucian* in **Hermotimus** adds headache:

**ἀλλὰ ἀνάγκη ἐν τῷ πελάγει διαφέρεσθαι ναυτιῶντα ὡς τὸ πολὺ καὶ δεδιότα καὶ καρηβαροῦντα ὑπὸ τοῦ σάου,…**

… he must be tossed about the sea, usually sick and frightened and with a bad head from the swell, …

In other classical texts, such as **Satyricon** by *Petronius*, symptoms of seasickness were considered relatively harmless, whereas *Plutarchos* in **Politika parangelmata** regarded them as a most distressing condition, in which one loses all self-control. *Plautus* stressed that seasickness may persist after sea travel, when leaving the ship and walking on stable land.

In ancient China, the source **Zhou hou bei ji fang** (300 AD) describes similar symptoms elicited as well during ship- or cart-travel (“Cart- and ship-influences”). Thus, afflicted individuals were described as suffering from agitation and pressure on the breast, headaches, and the urge to vomit.

### Susceptibility

The Greek classical authors observed that professional sailors reacted differently to the sea than did novices to sea travel. For example, *Ariston Chiou* notes in a passage of **Stoicorum veterum fragmenta**:

**Κυβερνήτης μὲν οὔτε ἐν μεγάλῳ πλοίῳ οὔτε ἐν μικρῷ ναυτιάσει, οἱ δὲ ἄπειροι ἐν ἀμφοῖν•**

Whereas a helmsman does not become seasick on a large ship or on a small boat, the inexperienced become seasick on both.

This description of unsusceptibility to seasickness, i.e., the result of repeated exposure to the stimulus, is now called habituation. Also *Livius* reported in his **Ab urbe condita** how many of Scipio’s soldiers, especially those who were not accustomed to the sea, had to spend some time recuperating after travel before entering battle:

**…necdum satis refectis ab iactatione maritima militibus,…**

… as his men were not yet fully recovered from the rigours of their sea voyage.

General illness was also regarded as a factor that augments susceptibility. *Cicero*, who suffered from seasickness as several passages in his **Epistulae** suggest, mentioned in the **Epistulae ad familiares** that it is not advisable to start a sea voyage when sick:

**Festinare te nolo, ne nauseae molestiam suscipias aeger et periculose hieme naviges**.

I would not have you to hurry yourself, lest you should have to suffer the agonies of sea-sickness in your feeble state, and lest a winter voyage should prove dangerous.

Lucian agreed in **Toxaris** that the ill will become seasick before anyone who is healthy and strong.

Ancient Chinese texts noted that children were particularly affected by cart travel and ship travel: they develop disorders of the heart, have headaches, and have to vomit. Susceptibility in children is described in **Zhou hou bei ji fang:**

(…)女子小儿多注车、注船、心闷乱、头痛吐(…)

many of the girls and boys [suffer] from cart-influence, ship-influence, pressure and disorder of the heart, headaches, and vomiting …

A similar concept is found in **Zhu bing yuan hou lun**:

嬖子小兒注車船候 signs of cart- and ship-influence in small spoiled children, or 無問男子女人，乘車船則心悶亂，頭痛吐逆，謂之注車、注船。特由質性自然，非關宿挾病也。

It makes no difference whether boy or girl, if they travel with the cart or the ship and this leads to pressure and chaotic feelings in the heart, if they have headaches and vomit, then this is called motion sickness. It is due to the actual nature of the individual and is not related to any past or other disease still present.

We did not find any hint in the classical Greek and Roman texts that infants were particularly susceptible to seasickness. Today, it is well known that infants below the age of 1–2 years usually do not become seasick, whereas their susceptibility increases significantly with age and reaches a peak between the ages of 7–12 years, before it declines to the susceptibility of adulthood (20, 21).

### Mental State/Psychological Predisposition

*Caesar* is one of several ancient Roman authors who emphasized that the prevailing mental state plays a role in developing seasickness. The satirical poet *Decimus Iunius Juvenal* also captures this aspect in a vivid and drastic scene in **Saturae** which evokes the suffering caused by seasickness without mentioning the word “nausea”. In his Sixth Satire, the well-meant advice of a friend is given to a groom: “Don’t get married.” All the vices of women are described, even a piece of scandal from the times of Emperor Domitian (51–96 AD): Eppia the wife of a senator has set off for Egypt with her lover, a gladiator. Although she was always seasick when traveling by water with her husband, her inner feelings will protect her during this sea crossing with her lover. Psychological factors made the crossing possible at all.

**…tunc sentina gravis, tunc summus vertitur aer. Quae moechum sequitur, stomach valet. Illa maritum convomit, haec inter nautas et prandet et errat per puppem et duros gaudet tractare rudentis**.

… the bilge-water then sickens her, the heavens go round and round. When she is following her lover, her stomach is robust; but as wife, she pukes all over her husband.

After the death of Caesar in 44 BC, the men of the second triumvirate (Octavian, Antonius, and Lepidus) took power. For Cicero, this meant that he was now considered an enemy of the state and had to flee the country. Traveling by ship as it was the fastest means of travel, Cicero suffered repeated bouts of seasickness until weary of life, he felt his will power broken. He could no longer flee, returned home, and took to his bed where he was assassinated by Anthony’s soldiers (43 BC). The Roman writer and rhetorician *Seneca Pater* describes Cicero’s fate in his **Suasioriae** from the first century AD, in which his aversion to the sea played a major role: forced to rest, he was assassinated,

**…unde aliquotiens in altum provectum cum modo venti adversi rettulissent, modo ipse iactationem navis caecovolvente fluctu pati non-posset, taedium tandem eum et fugae et vitae cepit, regressusque ad superiorem villam, quae Paulo post plus mille passibus a mari abest, ‘moriar’ inquit ‘in patria saepe servata’**.

… From there he traveled several times on the sea. However, soon the opposing winds forced him back; he could no longer bear the movements of the ship in the blindly whirling flood. Suddenly a great weariness to flee and even to live seized him. He turned back to his higher-lying villa, only about 1000 steps from the sea, saying “I will die in my fatherland, which I have often saved from annihilation.”

*Plutarch* too was aware of the influence of an individual’s emotions on his reaction to sea travel. *Having* extensively examined the various aspects of seasickness in **Aitiai physikai**, he was of the opinion that the psyche played a decisive role:

**ουδὲν οὖν ὄφελος τῆς ἔξω γαλήνης, ἀλλὰ ἡ ψυχὴ σάλον ἔχουσα καὶ θορυβομένη συγκινεῖ καὶ ἀναπίμπλησι τὸ σῶμα τῆς ταραχῆς**.

Thus the calm in their surroundings does them no good: their psychological tossing and being upset cause an accompanying disturbance in the body and infect it with their disorder.

Even quite bizarre behavior is said to result from seasickness. For example, *Lucian* in his description of Homer being transported by the ferryman Charon on a stormy river Styx in the Underworld has Charon witness Homer’s bout of seasickness. He is said to vomit some passages from **The Odyssey**.

In Medieval England, William Shakespeare (1564–1616) evokes the psychological effects of seasickness. Three years before the defeat of the Spanish Armada, Shakespeare settled in London. A writer famous for his awareness of the interests of his rulers and compatriots, he makes an unusual reference to seasickness in a rather surprising context in one of his most famous plays, Romeo and Juliet, finished in 1594. As Shakespeare is believed not to have traveled outside England, it is highly doubtful that he had first-hand experience of seasickness. Perhaps, he overheard descriptions of it in the pubs where the sailors of the main sea-faring country after Spain gathered or recycled them from another literary source. In the last act, last scene of the play before swallowing poison, Romeo takes leave of Juliet, who he mistakenly believes is dead, by saying

Come, bitter conduct, come, unsavoury guide!

Thou desperate pilot, now at once run on

The dashing rocks thy sea-sick weary barque!

Here’s to my love!

*[He drinks]* (22)

In his despair, Romeo identifies with the “weary barque” that itself is seasick. Its pilot (the poison) crashes it onto the rocks, destroying it and extinguishing his anguish at the loss of Juliet.

## Therapy

### Greek and Roman Approaches

#### Prevention

Some ancient authors reflected on how to prevent seasickness, most often emphasizing the importance of becoming accustomed to sea travels. The Greek author *Dieuches* who is cited by *Oreibasios* (**Oreibasiou iatrikai synagogai**) emphasized this as early as in the fourth century BC. Later, *Ariston* in the second century BC and *Aetios* in the 6th century AD in his **Logon iatrikon biblioi** also stressed the role of what one would today call habituation. *Juvenal*, however, disagreed. He believed that the psyche was the most important factor in the development of seasickness and thus did not think that habituation was possible—either one got seasick or did not. Other prophylactic advice given by classical authors were to fixate the shore (*Athenaios*) and avoid looking at the rough, open sea. Further, it was recommended to smell pleasantly fragrant plants like thyme, mint, or quince (*Dieuches*—cited by *Oreibasios* in **Oreibasiou synopsis pros Eustathion ton huion**). A passage from a herbal book from the fifth century AD, which is based on Greek sources (**Herbarius**, *Pseudo-Apuleius Platonicus*), recommends grinding up wormwood and mint, mixing it with olive oil and wine vinegar, and then frequently rubbing the mixture into the nostrils. The remedy “wormwood” for preventing seasickness was also recommended in a pharmaceutical didactic poem found in the **Macer floridus**, a work from the Middle Ages which is based on classical sources:

**Nausea non-poterit quemquam vexare marina, Antea commixtam vino qui sumpserit illam [absinthium]**.

Seasickness will not be able to torment him who drinks vermuth mixed with wine before setting off.

*Lucian* offered some advice in his **Hermotimus**, for example, to carefully observe the weather conditions and choose a reliable helmsman who is able to reduce upsetting ship motion by skillful steering maneuvers. Finally, *Soranos* at the beginning of the second century AD refers to nutrition, recommending fasting for 1 day before setting off on a sea voyage:

**…διόπερ ἀνατάσει χρηστέον. Καὶ γὰρ οἱ πλέοντες συστολῇ χρησάμενοι πρὸ μιᾶς ἡμέρας τὴν ναυτίαν οὐχ ὑπομένουσιν ἢ οὐκ ἐπι τοσοῦτον**.

… Thus fasting is the proper thing to do. In this way the sailors who fast one day before the voyage do not get seasick at all or only slightly so.

#### Therapeutic Recommendations

The above-mentioned preventive measures such as pleasant fragrances or wormwood are also used as therapy for acute seasickness; *Plinius*, for example, described in his **Historiae naturales** certain aromatic plants and herbs that can be taken to control nausea. Among these were mallow, fennel, wild cumin, rose petals boiled in wine, mint, or wormwood. Many of these plants can be found in other ancient sources like the **Macer floridus** (see above). *Athenaios* reports on divine help from the goddess Aphrodite who was believed to send pleasing aromas:

**'H δὲ ϑεὸς (προσφιλὴς γὰρ τοῖς Ναυκρατίαταις ἦν) αἰφνίδιον ἐποίησε πάντα τὰ παρακείμενα αὐτῇ μυρρίνης χλωρᾶς πλήρη ὀδμῆς τε ἡδίστης ἐπλήρωσεν τὴν ναῦν ἤδη ἀπειρηκόσι τοῖς ἐμπλέουσιν τὴν σωτηρίαν διὰ τὴν πολλὴν ναυτίαν γενομένου τε ἐμέτου πολλοῦ';**

The goddess – who was well-disposed to the inhabitants of Naucratis – immediately filled all the vessels that had been set before her with fresh myrtle, and the entire ship with a delicious scent, even though everyone on board had given up any hope of surviving, because they were so seasick, and there was a great deal of vomiting.

Dietary measures were also reported to be therapeutic by *Celsus*, a famous classical writer on medicine:

**Is vero, qui navigavit et nausea pressus est, si multam bilem emovuit, vel abstinere a cibo debet vel paulum aliquid adsumere. Si pituitam acidam effudit, utique sumere cibum, sed adsueto leviorem: si sine vomitu nausea fuerit, vel abstinere vel post cibum vomere**.

He too who on a voyage is troubled by seasickness, if he has vomited out a quantity of bile, should fast or take very little food. If he has spewed out sour phlegm, he may take food notwithstanding, but lighter than usual; if he has nausea without vomiting, he should either fast, or after food excite a vomit.

**Figure 3:**
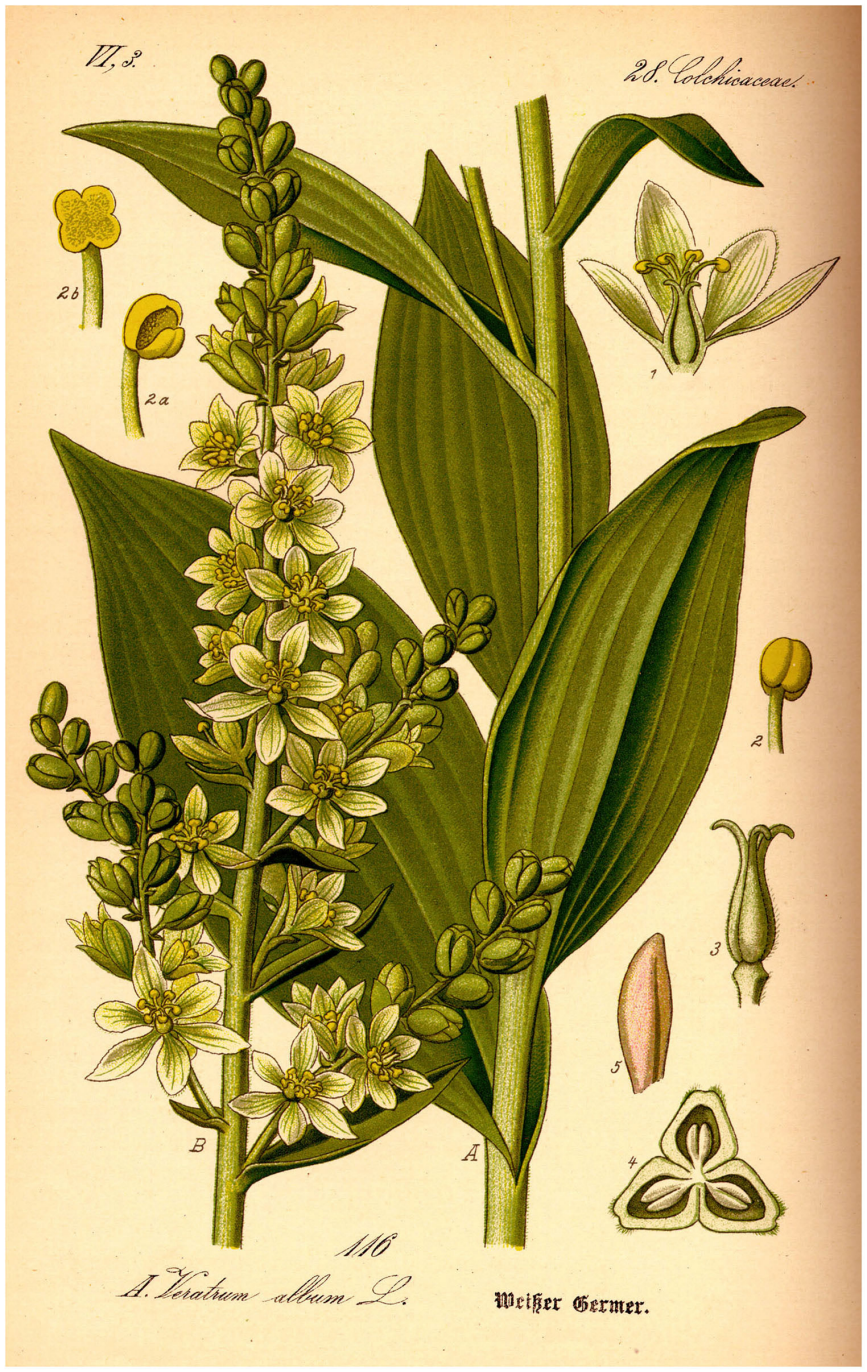
***Veratrum album* (white hellebore, έλλέβορος λευκóς) contains five alkaloids (rubijervine, pseudojervine, jervine, protoveratrine, and protoverarridine), all of which are poisonous**. Frequently used in antiquity as an emetic. *Hippokrates* (460–370 BC) already recommended it for seasickness in his **Aphorismoi**. The plant originated in the moderate climate zones of Asia and Europe, where it is still found in the Alps at heights of 700–2,000 m and more. It grows best on meadows and pastures, preferably in moist, nitrogen-rich soil, and reaches a height of 160 cm (https://de.wikipedia.org/wiki/Wei%C3%9Fer_Germer) (25).

Similarly *Oreibasios* reports that fasting or restricting oneself to a certain diet, namely dry or boiled lentils with mint or bread, pulverized in fragrant wine, can help. In case of continuous vomiting, sour honey was recommended. *Hippokrates*, who reported on seasickness in his **Aphorismoi**, recommended using the medicinal plant *Veratrum album* (white hellebore, “ἑλλἑβορος λευκός”), a violent gastrointestinal poison. The effective alkaloid-based emetic was widely used in antiquity, but therapeutic dosages differed from those currently used. *V. album* (white hellebore, Figure 3) contains five different alkaloids (rubijervine, pseudojervine, jervine, protoveratrine, and protoveratridine), which were first isolated by Salzberger (23). The habilitation thesis of Hahnemann (24) mentions that the contemporary white hellebore is thought to be identical to the plant referred to in classical texts. Its area of distribution includes the Alps, the Apennine mountains, and Eastern Europe. Several species of this genus also originated in Asia. The concentration of the alkaloid in the plant decreases with the increasing altitude of its habitat.

### Chinese Recommendations

The physician and author *Zhu Danxi* (1281–1358) is considered one of the most important physicians of his time. The following entry appears in paragraph 29, in the third chapter of **Danxi Xinfa**, his book on vomiting;

注船大吐,渴飲水者即死, 童便飲之最妙。(26)

Ship-influence with pronounced vomiting; those who have thirst and drink water will die, whereas it is very helpful to drink the urine of young boys.

A specific substance is discussed in **Ben cao gang mu** (Medical material organized by categories) from 1578. Its efficacy for cart- and ship-influence 注车注船 zhuche zhuchuan is also assessed as part of a definite remedy.

Reference is made here to the source **Zhou hou bei ji fang**.

注车注船:凡人登车船烦闷,头痛欲吐者。

Cart- and ship-influence describes the phenomenon of suffering from agitation and pressure on the breast, headaches and the urge to vomit when traveling on a cart or a ship.

An entry in **Yan fang xin pian** (New collection of tested remedies) from the 17th/18th century discusses the same phenomenon under the concept of ship-dizziness and cart-dizziness l 晕船晕车 yun chuan yun che: “As regards head-dizziness (touyun) and vomiting caused by sitting on a ship, earnestly pray at the start of the trip to the palace of the goddess of sailors or to the place of the river and water spirits; this will bring peace. Or drink the urine of young boys, this has a wondrous effect, or take white sand-sirup, that’s also effective. Another remedy is to collect water that drops from a long bamboo stick into a bowl, add this to boiling water, and drink the mixture; this too is effective. Another remedy: If you go on a ship, take some earth from the middle of the kitchen hearth and hide a piece under your hair; don’t tell anyone, this gives peace. Or write the character for dirt/earth on your palms. If you then go on a ship, you will have nothing to fear. Earth can protect you from the meaning (threat) of water.”

## Historical Evidence of Military Impact

### Roman Period

*Titus Livius* (59 BC–17 AD), a historian from Patavium (today known as Padua), spent a large part of his life in Rome. In his historical opus **Ab urbe condita** (Buch XXI, 26), it is obvious that the troops of Scipio who were unaccustomed to sea travel became seasick and needed time to recover. This is described in an episode from The Punic War (218–201 BC). Hannibal was marching with his troops in the direction of the estuary of the Rhone. To oppose him, Publius Cornelius Scipio Africanus Maior was moving 60 of his warships along the coast of Etruria and Liguria and along the regions of the Saluer toward Massilia, modern-day Marseille. Unfortunately Scipio’s soldiers, unaccustomed to sea travel, had become seasick. It was feared that Hannibal could begin crossing the Rhone at any moment. Fortunately, in the end, however, Scipio was able to allow his soldiers to take a rest to sufficiently recover: they did not have to immediately engage in a great battle with Hannibal upon landing.

**…necdum satis refectis ab iactatione maritima militibus,…**

… as his men were not yet fully recovered from the rigours of their sea voyage.

In the commentaries to his book **De bellum civile**, *Gaius Julius Caesar* (100–44 BC) reports how his troops crossed over from Brandisium (48 BC) (contemporary Brindisi) to the Greek mainland. The stormy weather swept some of the soldiers onto the shores, which were patrolled by the enemy. Exhausted by seasickness, they surrendered and were put to death. Others were nevertheless able to mobilize energy for the ensuing battle.

**…tirones enim multitudine navium perterriti et salo nausiaque confecti iureiurando accepto nihil iis nocituros hostes se Otacilio dediderunt; qui omnes ad eum producti contra religionem iusiurandi in eius conspectu crudelissime interficiuntur. at veteranae legionis milites, item conflictati et tempestatis et sentinae vitiis, neque ex pristina virtute remittendum aliquid putaverunt,…**

… For the recruits, terrified by the number of ships and exhausted by the rough water and seasickness, after receiving a solemn pledge that the enemy would do them no harm, surrended themselves to Otacilius; and all of them, when brought to him, were most cruelly massacred before his eyes in violation of the sanctity of his oath. But the men of the veteran legion, though equally distressed by the discomforts of the storm and the bilge-water, considered it their duty to relax nothing of their pristine valour, …

In this context, it is interesting that another medical condition, namely the fear of heights, also influenced historical warfare, for example, the conquest of Carthago Nova reported by *Titus Livius* in his History of Rome (**Ab urbe condita**, libri CXLII).: “But neither the men nor the projectiles nor anything else defended itself as well as the city wall. The few scaling ladders that were as high as the wall were flawed, and the taller the ladder was, the more flawed it was. For this reason they broke under the weight of the soldiers. Those who had just reached the highest point were not able to escape, while others pressed up from below. Several plunged down to the ground, while the ladders were still standing, since the heights (altitudo) had veiled their eyes with dizziness (caligo).” Ultimately, the high walls did not prevent the conquest of Carthago Nova (17).

### Chinese Antiquity and the Battle of Red Cliff

Chinese society has long valued the past, especially ancient history and stories of former times. The Battle of Red Cliff (also known as the Battle of Chibi) took place in 207 AD. It is the subject of poems and films about the heroic battle to unify the empire. The narrative describing the battle suggests that the swaying motion of the ships was perceived as causing illness and an absence of well-being; however, none of the known terms indicating seasickness appear. Two works contain information about the story and the battle. The first report, “Records of the Three Kingdoms” (San guo zhi) from the 3rd century, is the officially authorized history book, covering the years 184–280. The second book, “Romance of the Three Kingdoms” (San guo yan yi), is a popular novel about ancient history (27). Dating from the 14th century, it is considered one of the four classical novels of ancient China, and thus the crowning achievement of Chinese narrative art.

The novel gives a more fictionalized and dramatized report on the famous battle, hinting that health problems arose from the continuous swaying of the ships. Commander Cao Cao and his soldiers are northerners and unfamiliar with warring on ships. The enemy exploits this by having an alleged friend of Cao Cao suggest an apparent solution: to increase the stability of the ships by tying and nailing them together, and thus prevent the soldiers from becoming sick. Cao Cao follows the advice and acts accordingly, with the result that the enemy successfully sets fire to the entire fleet, in the ensuing battle. The following fateful dialog takes place:

While conversing with commander Cao Cao an officer named Tong asks: “May I inquire whether there are any good physicians among the soldiers (liang yi)?” Cao responds’ “Why is that important?” Tong replies: “Many of the soldiers are sick, and good physicians are needed to treat them.” It was actually true that Cao’s troops were not accustomed to the local climate and began vomiting; many died. After Cao had thoroughly considered the situation, he suddenly asks Tong how to avoid this? Tong replies: “In the middle of the Great River (Jiangzi) the water rises and falls, the wind continually creates waves, the soldiers from the north are not used to traveling by ship. This rolling and lurching causes illness. It would help to connect the large and the small boats together, 30 or 50 per row, prow to stern, with iron hoops or to put broad planks on them so that men and horses can cross over them. Then regardless of how the wind, waves, and tides danced, what would there be to still fear?” It is clear that the motion of the river is considered to be the cause of the soldiers’ weakness and sickness.

### Medieval England and Defeat of the Spanish Armada in 1588

**Figure 4:**
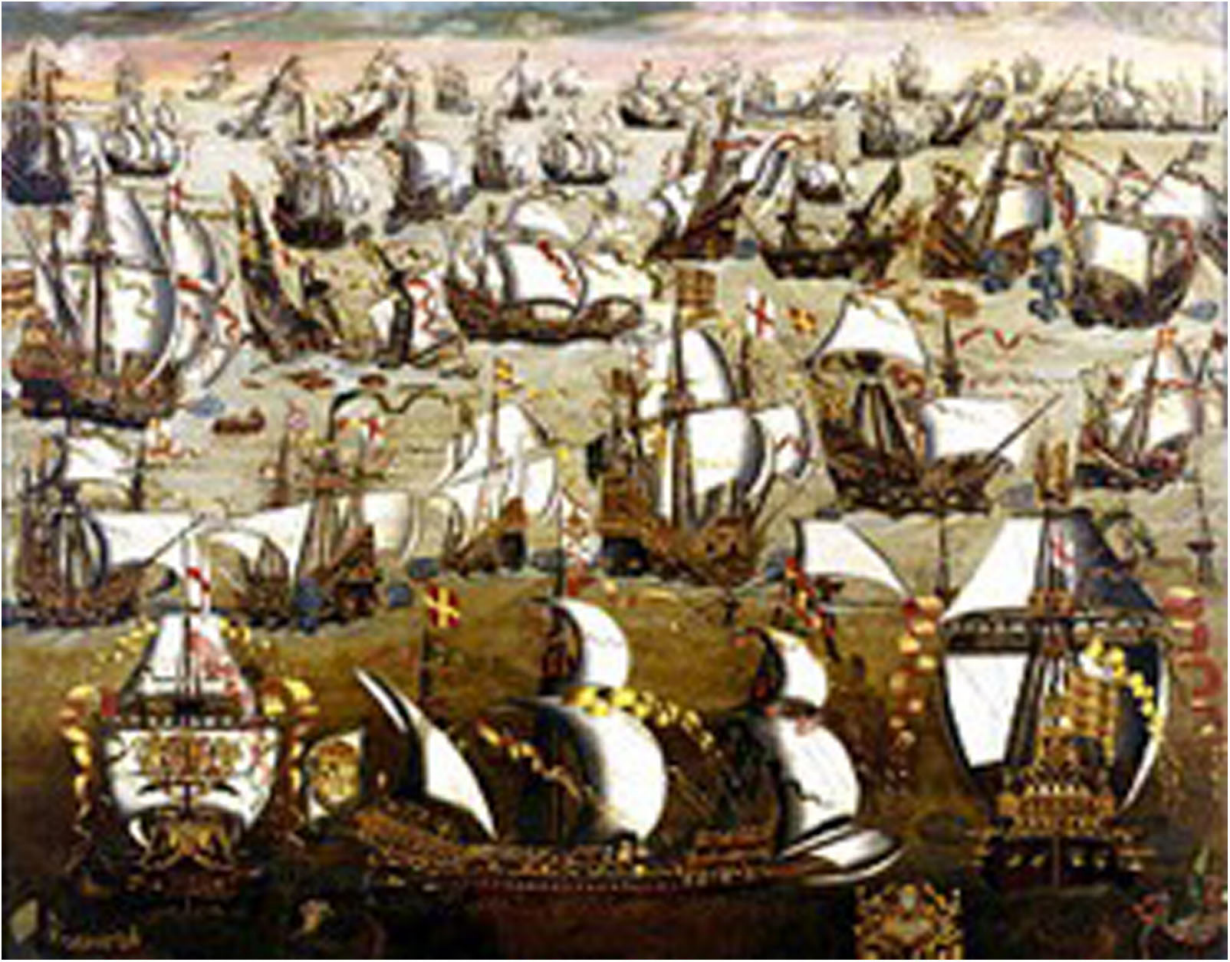
**Spanish Armada**. Unknown contemporary artist painting before 1700 AD. The defeat of the Spanish Armada in 1588 was the beginning of English dominance of the sea. In foreground English ships are shown surrounding and attacking Spanish galleons. Painting in National Maritime Museum, London (https://de.wikipedia.org/wiki/Spanische_Armada#/media/File:Invincible_Armada.jpg) (28).

A naval battle probably equal in importance and heroic qualities to the Battle of Red Cliff for the Chinese was for the English the battle against the Spanish Armada off the shore of England in 1588 (Figure 4). A lot was at stake—the rule of Spain over England and the reversal of the reformation that had been bought at such high costs. The English saw the defeat of the Spanish as a turning point in their history. Not only were Spain’s plans to conquer England dashed but it also lost its reputation as the world’s main naval force. Afterward, naval power was concentrated in the north Atlantic and was primarily in the hands of the English and the Dutch fleets.

Many factors played a role in the defeat of the Spanish. A major one was that King Philipp II of Spain failed to consult his naval experts and had proposed a plan that at the time was considered unpromising if not disastrous. He assumed his naval forces were sufficient and sent around 160 Spanish galleons, mostly massive and old ships. These proved too unwieldy to counter the smaller, faster ships of the English. While the English had advanced, very effective long-range cannons, the Spanish had nothing comparable and were poorly armed. Their battle strategy also differed. The English specialized in fireships, setting afire old galleons loaded down with gun powder and wood that were then directed toward the Spanish. The slow movement of the Spanish ships often meant they could not escape, falling victim to the conflagration. The strategy of the Spanish had relied on forces from Parma, which did not arrive in time due to Dutch interference. No other alternative plan had been made. Luck also played a role. A storm arose, and the choppy waters of the channel and the open sea to which the Spanish were unaccustomed, became even more threatening. Trying to flee, they crashed upon the shore.

Incredibly, Spanish manpower consisted of three times as many foot soldiers as sailors, for the plan had been to fight mainly on land, not on water. The man in charge of these forces, Don Alonzo Perez de Guzman el Bueno, the Seventh Duke of Medina Sidonia, had no experience with naval battles and was often seasick. He had tried to refuse the appointment by openly stressing his lack of experience to Phillip II, but he was not heard (29). Many of his soldiers likewise had never fought on water before and were not only seasick but also afflicted with typhus. In contrast, the English had very experienced commanders, Lord Howard of Effingham and his second-in-command Sir Francis Drake. The Spanish especially feared Francis Drake, who was known as “the Dragon,” an erroneous translation of his name. While many considered him a pirate, he proved his sailing and commanding skills (30). Only half of the original Spanish forces made it back home alive (31).

### Napoleon and His “Camel-Sick Corps”

**Figure 5:**
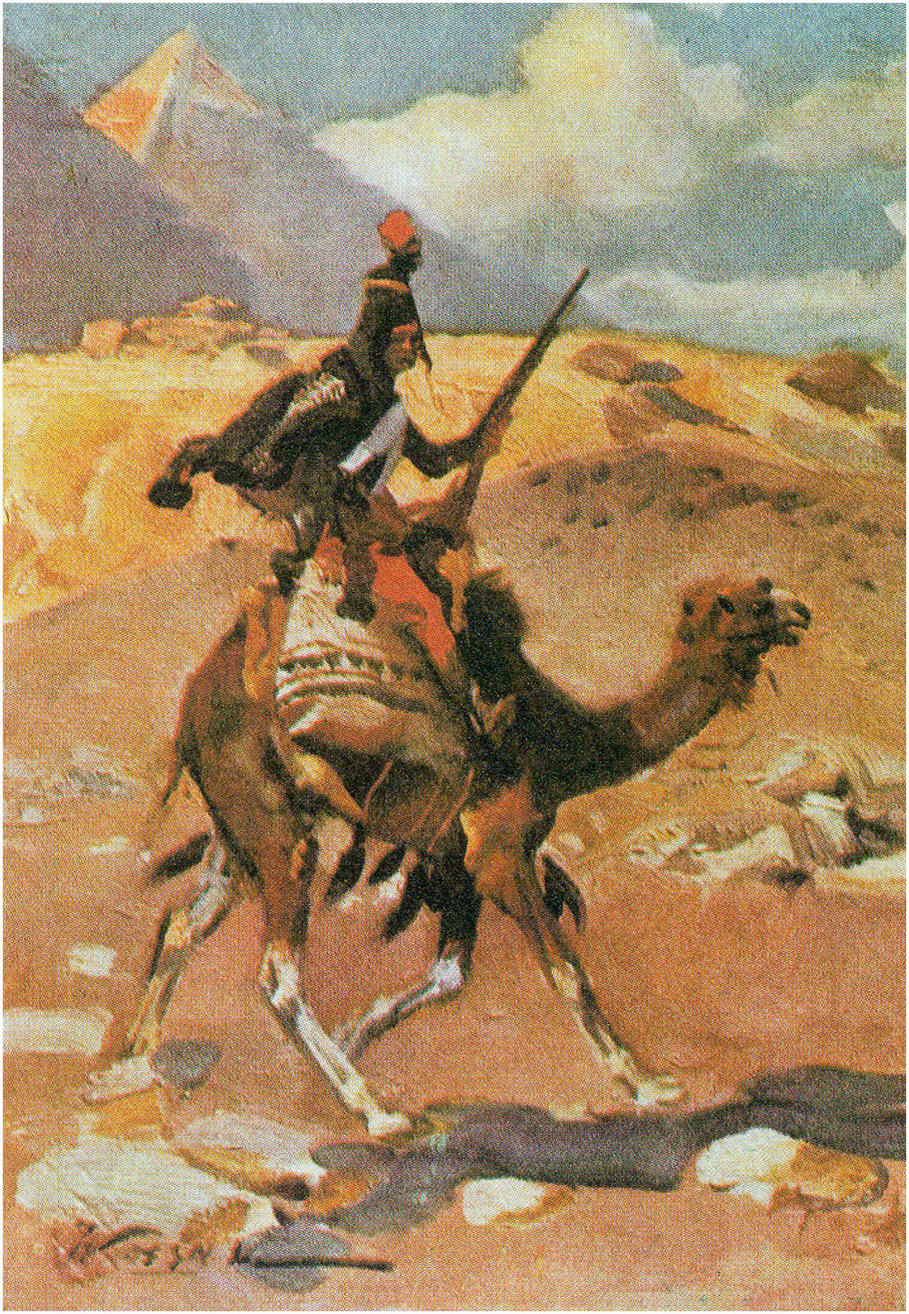
**Napoleon and camel-sickness**. A soldier of Napoleon’s camel (dromedar) regiment, initiated by Napoleon in 1799. Interestingly, the soldiers were considered infantry, because they dismounted to fight. Those who couldn’t adapt to the movements of the “ship of the desert” became seasick, proving rather ineffective combattants (Un soldat du regiment dromedaries, par Wojciech Kossak, 1912; https://fr.wikipedia.org/wiki/Régiment_des_dromedaires#/media/File:WKossak028.jpg).

A curious footnote to the history of war and “seasickness” is connected with Napoleon. While the military significance was not considered important, the appearance of seasickness in the desert was unusual. In his first military campaign against Egypt (1798–1799), General Napoleon conceived the idea of creating a camel corps. He supposedly had become aware of the camel’s suitability for transportation quite early. Although riding a camel did cause him some nausea, it was not so considerable as to prevent him from using one himself. Indeed his special camel was embalmed when it died of natural causes and is said to still be on exhibit in the African museum in Paris. In January 1799, Napoleon instituted the dromedary regiment which saw action after an attack of the Beduins (Figure 5).

Camels had several advantages over horses, namely, they did not have to drink so often and were adapted to the harsh conditions of the desert. They could easily travel for hours and outrun as well as outlast enemies on horses. One major drawback, however, was that soldiers who were susceptible to motion sickness could become “seasick” on this “ship of the desert” and not be able to engage in battle (32). The corps saw action for a period of three years (1798–1801). It was disbanded after France withdrew from Egypt following losses to the British.

## Author Contributions

DH and TB conceived and designed the study, analyzed and interpreted the classical texts, and wrote the paper. JB analyzed and interpreted the classical texts, and edited the manuscript.

## Conflict of Interest Statement

The authors declare that there are no conflicts of interest and that there exist no financial or other relationships that have influenced the work.

## Footnotes

^1^The names of the ancient authors are written in italics; their writings and citations from them are written in bold. All other authors and titles appear in a normal font. Throughout the text, we have either quoted directly the original sources and their translations (quotation marks) or we have paraphrased them for more immediacy.
